# Ovarian steroid cell tumors: what do we know so far?

**DOI:** 10.3389/fonc.2024.1331903

**Published:** 2024-01-30

**Authors:** Christina H. Wei, Oluwole Fadare

**Affiliations:** ^1^Department of Anatomic Pathology, City of Hope National Medical Center, Duarte, CA, United States; ^2^Department of Pathology, University of California, San Diego, La Jolla, CA, United States

**Keywords:** ovarian steroid cell tumor, hyperandrogenemia, ovarian neoplasm/diagnosis, sex cord stromal tumor, virilization, ovary

## Abstract

Steroid cell tumors (SCT) of the ovary are rare, which has limited advances in the understanding of this enigmatic neoplasm. In this review, we summarize currently known clinicopathologic information on SCT. SCT are frequently hormonally active, leading to elevated serum and/or urine levels of androgenic hormones or their metabolites, and associated symptomatology, including virilization. The reported age at diagnosis is broad and has ranged from as young as 1 year old to 93 years old, although most patients were between ages 20 and 40 years. Most tumors are stage I and unilateral. The tumors are usually well circumscribed with a solid or solid to cystic cut surface. The tumors in one series reportedly ranged in size from 1.2 to 45 cm (average 8.4 cm). MRI is a useful imaging modality, typically showing a well delineated mass with contrast enhancement and lipid content on T2 and T1 weighted images, respectively. Microscopically, SCT display polygonal to epithelioid cells with abundant eosinophilic to vacuolated/clear cytoplasm and display an immunoprofile that is consistent with sex cord-stromal differentiation. Most cases are benign, without any recurrences after primary resection, but a subset – probably less than 20% of cases –are clinically malignant. Pathologic criteria that can specifically predict patient outcomes remain elusive, although features that correlate with adverse outcomes have been proposed based on retrospective studies. The molecular characteristics of SCTs are similarly under characterized, although there is some evidence of an enrichment for hypoxia-signaling gene mutations in SCT. In malignant SCT, the tumors generally show greater global genomic instability, copy number gains in oncogenes, and occasional BAP1 mutation. Future studies involving multi-institutional cohort and unbiased molecular profiling using whole exome/transcriptome sequencing are needed to help advance our molecular understanding of SCTs.

## Introduction and historical evolution

In the 5^th^ edition of the World Health Organization (WHO) classification of female genital tumors, steroid cell tumor (SCT) is defined as “an ovarian parenchymal tumor comprised of steroid cells.” (1) This simple definition is a reflection of the current understanding of this rare and enigmatic neoplasm. Historically, it has long been recognized that a subset of ovarian tumors that are associated with virilization are exclusively comprised of cells that closely resemble steroid hormone secreting cells, including the adrenocortical cortical cells, Leydig cells, and lutein cells (2). For several decades, different authors applied a variety of terms to these lesions, including androblastoma diffusum, arrhenoblastoma, Leydig cell tumor, adrenal or adrenocortical tumor, adrenal rest tumor, adrenal-like tumor, stromal luteoma, lipoid or lipid cell tumor, virilizing or masculinizing lipoid/lipid cell tumor, ovoblastoma, masculinovoblastoma, sympatheticotropic tumor, hilus cell tumor, and hypernephroma/hypernephroid tumor (2–7). The 1st edition of the WHO classification of ovarian tumors (1973) included Leydig cell tumors and lipoid cell (or lipid cell) tumors as separate entities, with the latter defined as a tumor comprised of one of the aforementioned steroid hormone secreting cells, but which “cannot be identified specifically as any one of the three types.” (6) Given that many neoplasms of this class are comprised of tumor cells that contain no significant amounts of intracytoplasmic lipid, the term “lipoid or lipid cell tumor” was not ideal, and ultimately led to its replacement by “Steroid cell tumor”, a term that was initially proposed by Dr. Robert E Scully in 1979 (8) as a better descriptor for the group of tumors that included stromal luteoma (9), Leydig cell tumor (10) and tumors in this class that could not be classified as either of these 2 entities - steroid cell tumor not otherwise specified (SCT NOS) (11). These 3 entities were thought to comprise 20%, 20% and 60% of steroid cell tumors respectively. A Leydig cell tumor is a benign, typically androgen producing tumor that is usually confined to the ovarian hilum and which commonly shows cytoplasmic Reinke crystals (1, 6). Stromal luteomas were initially conceptualized as benign, small, ovarian cortex-confined neoplasms that were mostly seen in postmenopausal patients (11, 12). Patients most frequently presented with abnormal vaginal bleeding that was probably attributable to hyperestrogenism (11, 12). Although stromal luteomas were thought to display distinctive clinicopathologic features (13, 14), starting with the 4^th^ edition of the WHO classification of ovarian tumors (2014), stromal luteoma ceased to be recognized as a distinct entity (15). Tumors that were previously classified as stroma luteoma and SCT NOS were both subsumed under the SCT (15), and the latter has remained the preferred terminology for this tumor (1). SCTs are rare, with fewer than a thousand cases reported in the literature to date. This rarity has limited advances in the understanding of this enigmatic neoplasm. In this review, we summarize currently known clinicopathologic information on SCT.

## Clinical and radiologic presentation

SCT are frequently hormonally active, leading to elevated serum and/or levels of androgenic hormones and their metabolites (11, 16, 17). In a subset of cases, ovarian SCT can induce ACTH secretion, leading to co-presentation of Cushing syndrome (18–21). Symptomatology is often related to androgenic excess, including virilization, hirsutism, balding, deepening of voice, acne, and clitoromegaly (11, 16). Overall, the most common initial manifestation in one series was virilization (41%), although 6.3% had estrogenic manifestations. In additional to symptoms related to androgen excess, there are age group-specific presentations. For example, in pediatric population, children may show isosexual precocious puberty (22). In child-bearing age group, women present with irregular menstrual cycles or infertility (23). In post-menopausal women, vaginal bleeding may occur (24, 25). In most cases, SCTs present as an unilateral ovarian tumor (11). However, it has been estimated that 6% of patients present with bilateral ovarian SCTs (11, 26). A subset of SCTs are malignant (11, 27)., and malignant SCTs has been reported in females as young as 4 years old (28). Malignant SCT presents with extra-ovarian disease, often involving the retroperitoneum, mesentery, omentum, and other intraabdominal organs such as colon (29). Distant metastasis includes the vertebral bone and brain (30). A rare case of malignant ascites from peritoneal dissemination has also been reported (31).

The age at diagnosis is broad, ranging from as young as 1 year old to 93 years old, but generally between 20s-40s. In one series (11), the average age was 43 years (range 2.5-80 years), and in one review of the literature, the median age was 33.5 years (range 3-93) (16). Accordingly, a significant number of ovarian SCT occurs in the pediatric population, wherein the tumors may initially be misdiagnosed with congenital adrenal hyperplasia, which may exhibit similar clinical symptomatology (32, 33). Along the same vein, women of reproductive age with ovarian SCT may be misdiagnosed with polycystic ovarian syndrome (PCOS) - a much more common hormonal disorder in this age group (34). Another critical point to underscore is that while the majority of the cases present with a unilateral ovarian mass (size ranging from 1.2-45 cm), smaller lesions may be missed by modern imaging techniques such as MRI, leading to underdiagnosis of ovarian SCT (35). Indeed, an integrative clinical, radiologic, and biochemical workup is necessary to achieve optimal screening. On rare occasion, for diagnostically occult cases, therapeutic oophorectomies has been performed to exclude the possibility of ovarian SCT (36). In general, MRI has the most specificity for a SCT, which typically demonstrates a well-defined solid mass. Key characteristics include contrast enhancement on T2-weighted image (37), and demonstration of lipid content on T1-weighted image with signal drop between pre-contrast T1-weighted opposed phase and T- weighted in phase images (38). On balance, clinical presentation of virilization, increased serum testosterone level, and presence of a lipid-containing ovarian mass on MRI should raise the differential diagnosis of an ovarian SCT.

Ovarian SCT can occur in patients with germline mutations *in FH, VHL, and APC* genes. The most frequently reported cancer predisposition syndrome associated with ovarian SCT is VHL. There are 5 reported cases of SCT arising in VHL patients in the literature, four are unilateral on presentation and one is bilateral (39, 40). The onset age ranged from 16 to 46 years old (39). There is only one case report of a patient with germline *FH* mutation. This patient presented with asynchronous bilateral ovarian SCT, initially at age of 22 (left ovary, 2 cm), and later at 31 years old (right ovary, 6.3 cm) (41). There is also one case report of a benign, unilateral ovarian SCT in a 47-year-old woman with familial adenomatous polyposis syndrome (42).

## Macroscopic, microscopic, and immunohistochemical features

**Figure 1:**
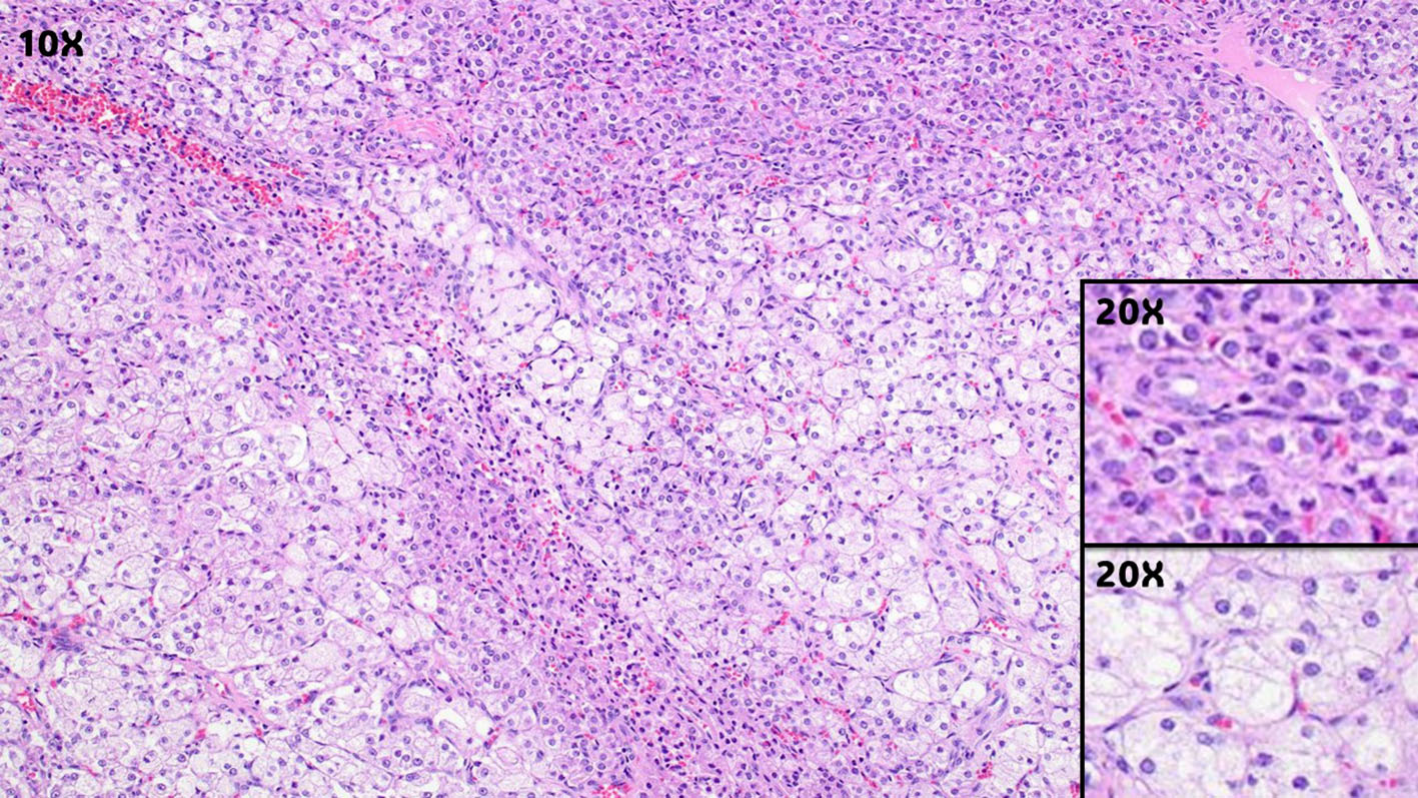
Ovarian steroid cell tumor, showing solid sheets of epithelioid to polygonal cell with eosinophilic to clear cytoplasm (Magnification, 10X). The insets show higher magnification of the tumor cells with eosinophilic cytoplasm (top inset, magnification 20X), or clear/vacuolated cytoplasm (lower inset, magnificent 20X).

In one series of 63 cases, 51, 4,7 and 1 case(s) were stage I, II, III, and IV respectively (11). 94% were unilateral and 6% bilateral (11). The tumors reportedly ranged in size from 1.2 to 45 cm (average 8.4 cm); 65% were described as well circumscribed and a smaller subset as encapsulated (11). Most were described as having a solid cut surface, with smaller subsets being solid to cystic or entirely cystic (11). The tumoral cut surfaces were mostly yellow, or in a minority of cases, brown, tan or gray white (11). Calcifications, hemorrhage or necrosis may be grossly observed. Microscopically, SCT comprises a proliferation of polygonal to epithelioid cells with abundant eosinophilic to vacuolated/clear cytoplasm (**Figure 1**). The nuclear and nucleolar size may vary from case to case or within a given case, as may the level of nuclear pleomorphism. The cells are arranged in sheet-like to nested patterns, separated by a delicate vascular network. Most cases have a low mitotic index, but this may vary as well. Necrosis, lymphovascular invasion, zones of hypercellularity, stromal hyalinization, lipid droplets, vague spindling and/or hemorrhage may be seen. Significantly, no Reinke crystals are present (a defining feature of Ledyig cell tumor). The immunoprofile of SCT is consistent with sex cord-stromal differentiation, with >80% expressing inhibin-A, SF1 and calretinin (41,42). A subset of SCT variably (30-70%) demonstrate positivity for CD99, androgen receptor, Melan A, estrogen receptor, progesterone receptor, SMA, CD10 and pancytokeratins (41,42,43). SCT do not express WT1 or epithelial membrane antigen (43).

## Molecular pathogenesis

Three molecular studies have been reported on SCT (27, 43, 44). One of aforementioned studies included a “metastatic” ovarian Leydig cell tumor, which likely present a steroid cell tumor (44). Overall, there appear to be few, if any, pathognomonic recurrent mutations for SCT. This contrasts with other types of ovarian sex cord stromal tumors, such as Sertoli-Leydig cell tumor, Sertoli cell tumor of pure-type, sex cord-stromal tumors with annular tubules (SCTAT), adult granulosa cell tumor (AGCT), and juvenile granulosa cell tumor (JGCT). In one series, 60% of the Sertoli-Leydig cell tumors was found to have *DICER1* mutation, and some occurred in the setting of germline *DICER1* mutation (45). A subset of SCTAT and pure-type Sertoli cell tumor cases arise in association with germline *STK11* mutation that causes Peutz-Jegher syndrome (46). Interestingly, SCTAT occurring in context of syndromic germline STK11 mutation have improved outcomes compared to sporadic/non-syndromic patients (47). Over 95% of AGCT demonstrates recurrent somatic *FOXL2* mutation (48). JGCT may occur in the setting of Ollier disease and Maffucci syndrome, or somatic mosaic mutations in *IDH1* and *IDH2*, and somatic copy number changes in *AKT* (45).

In ovarian SCT, a more heterogeneous profile of genetic mutations has been reported, including *BAP1, FH, TP53, CTNNB1, CASP10, HIF1A, SRC, FOXO4, HOXA13, LHCGR, VHL, IDH2, SDHB*, and *BRCA2* (27, 43, 44). Most are missense mutations, except for *BAP1* and *FOXO4*, which are frameshift mutations (27, 43), and *SRC*, which is an in-frame deletion (43). In one of the studies, enrichment of hypoxia-associated gene mutation (*HIF1A*, *VHL*, *SDHB*, *SRC*, *IDH2*, and *FOXO4*) was observed in a retrospective case series of 5 benign and 2 malignant SCT patients (43). Interestingly, SCT has been reported in patients with germline mutation in *VHL* (39), suggesting a correlation between hypoxia signaling pathway in the tumorigenesis of SCT. Wnt signaling pathway is another implicated pathway dysregulated in SCT, since somatic *CTNNB1* mutation and biallelic *APC* loss molecular events have been reported in SCT (27, 42).

For malignant SCTs, we found a total of six malignant SCT with molecular information, reported by three independent studies (27, 43, 44). The molecular findings are not entirely consistent between series. However, two general observations were seen. First, malignant SCTs exhibited more global genomic instability by copy number analysis. This is supported by the identification of copy number gain in *MDM2* and CDK2 *genes*, *ATRX* rearrangement, and copy number amplification in *NPM1*, *DCM1*, and *SS18* genes (27, 44). However, it is important to note that these genes are sporadically reported and are not consistently found in all malignant SCT cases. More likely, these identified amplification and structural rearrangement events are passenger events secondary to global genomic instability. Second, *BAP1* mutation was found in two of the six malignant SCT cases sequenced to date, reported independently by two groups (27, 44). The mutation genotypes for *BAP1* were p.K453fs and p.S126Rfs*61 (personal communications with Dr. Vranic and Dr. Bennett). Interestingly, *BAP1* mutation has not been reported in benign SCTs to date. Other mutations found in malignant SCTs included *HIF1A* and *SDHB* (44).

Although the data is limited, other possibly negative molecular findings include: (1) The type of gene mutations does not appear to be correlated with the number of adverse histologic risk factors (27), and (2) Microsatellite instability was not identified in any tested sample, suggesting that SCTs are likely not hyper-mutated tumors (43, 44).

On balance, the malignant cases are genetically more unstable, characterized by global chromosomal number aberration, with occasional *BAP1* mutation. However, readers are cautioned to avoid overgeneralizing these findings due to the small sample size. The genomic profile of benign and malignant SCTs is still relatively under-characterized, secondary to limited samples of this rare tumor type, and the selective use of cancer gene panel assays to profile their genomic makeup in the published studies (27, 43). Indeed, some noncancer-related genes, such as metabolic or hormonal-related genes, may be important for the development or prognostication of SCTs. Future studies with larger sample size, and the use of more advanced, unbiased molecular techniques, such as whole exome and transcriptome molecular profiling, will ultimately provide a more comprehensive molecular profile of SCT.

We found one functional molecular study of SCT in the literature (49). Using telomerase repeat amplification protocol (TRAP) assay, this study showed intact telomerase activity in a malignant SCT. In a retrospective series of sex cord-stomal ovarian tumors, Dowdy et al. demonstrated that telomerase activity has a 94% specificity for malignancy. In the same study, none of the benign sex cord-cord-stomal ovarian tumors showed telomerase activity. The prognostic significance of telomerase activity in SCT, particularly in distinguishing benignity from malignancy warrants further investigation (46).

## Patient outcomes and possible pathologic predictors

Most reported cases of SCT have been clinically benign without recurrences or death from disease following the primary resection of the tumor (11,16). In a recent review of the literature, Lin et al. found post-resection disease recurrence or progression occurred in 17.86% of cases, with a median tumor-free interval of 23 months (16). The authors noted that recurrences seemed to be associated with patient age, with a recurrence rate of 11.43% for patients aged 40 years or younger, and 28.57% for those older than 40 years, and no patients younger than 20 years of age reported with recurrence or progression. In the series of Mendoza et al, approximately 14% of cases were malignant (27). In the series of Hayes and Scully, most of which were consultation or referral cases, approximately one third of cases were clinically malignant (11). Overall, our impression is that the malignancy rate is probably less than 20%. The authors noted that the best pathological correlates of malignant behavior were: the presence of two or more mitotic figures per 10 high power fields (92% malignant); necrosis (86% malignant); a diameter of 7 cm or greater (78% malignant); hemorrhage (77% malignant); and grade 2 or 3 nuclear atypia (64% malignant) (11). In one case series, although all malignant SCTs demonstrated at least 4 atypical features, at least one atypical feature was present in benign cases as well (27). Thus, pathologic features that are specifically predictive of behavior have not been conclusively defined, although the data suggests that there may be features that correlative with adverse outcomes. A combination of pathogenomic classification may improve our ability to classify the prognosis of SCTs with atypical features.

In malignant cases, patients may either present with advanced extra-ovarian disease or recur after surgery. The disease recurrence timeline is variable and can recur within months or as long as 17 years after initial diagnosis and surgery, even in stage IA cases (27, 43). Metastatic SCT typically presents with intra-abdominal and retroperitoneal metastases, and on rare occasions, ascites. The clinical course for malignant SCTs are generally guarded, and most succumb to the disease 6-44 months following the diagnosis (11, 16, 27, 29, 30). However, as previously noted, recurrences may occur many years after primary resection. SCTs are generally insensitive to chemotherapy (29, 50). Rare case reports of disease control with a GnRH agonist have been reported (51, 52). In benign cases, the serum testosterone level generally normalizes within days or weeks following surgical resection of SCT (16). Successful pregnancy is achievable following surgery, usually within 1 year of tumor removal (53, 54). Virilization and hirsutism are usually resolved within a year of surgical tumor removal. This underscores the importance of early detection and surgical management of SCT. However, to prevent overtreatment the readers are cautioned that increased use of prenatal ultrasound has led to increased detection of asymptomatic ovarian masses (55). Most adnexal masses detected during gestation are benign and functional (55). The most common sex cord stromal tumors detected during gestation are granulosa cell tumor (22%), thecoma (18.6%), and Sertoli-Leydig tumors (8.5%) (56). Fortunately, greater than 70% of sex cord stromal tumors found during pregnancy result in live births (56).

## Summary and conclusions

Ovarian SCT are rare, with fewer than a thousand cases reported in the literature to date. SCT patients frequently display evidence of androgenic excess, with elevation in plasma testosterone level. A subset of SCT occurs in patients with germline mutations in *VHL, FH,* and *APC* genes. While most SCT are benign, a small subset are malignant and recurrences may occur many years after primary resection of an apparently localized tumor. Pathologic criteria that can specifically predict patient outcomes remain elusive, although features that correlate with adverse outcomes have been proposed based on retrospective studies. The molecular characteristics of SCTs are still under characterized, due to rarity of this entity. However, a few key observations have been made, including an enrichment of hypoxia-signaling gene mutations. In malignant SCT, the tumors generally show greater global genomic instability, copy number gains in oncogenes, and occasional *BAP1* mutation. Future studies involving multi-institutional cohort and unbiased molecular profiling using whole exome/transcriptome sequencing are needed to help advance our molecular understanding of SCTs.

## Author contributions

CW: Conceptualization, Data curation, Writing – original draft, Writing – review & editing. OF: Conceptualization, Data curation, Formal analysis, Investigation, Methodology, Supervision, Visualization, Writing – original draft, Writing – review & editing.
